# Initiation Timing of Continuous Interscalene Brachial Plexus Blocks in Patients Undergoing Shoulder Arthroplasty: A Retrospective Before-and-After Study

**DOI:** 10.3390/jpm12050739

**Published:** 2022-05-01

**Authors:** Ha-Jung Kim, Hyojune Kim, Kyoung Hwan Koh, In-Ho Jeon, Hyungtae Kim, Young-Jin Ro, Won Uk Koh

**Affiliations:** 1 Department of Anesthesiology and Pain Medicine, Asan Medical Center, University of Ulsan College of Medicine, 88, Olympic-ro 43-gil, Songpa-gu, Seoul 05505, Korea; alexakim06@gmail.com (H.-J.K.); ingwei2475@gmail.com (H.K.); yjro@amc.seoul.kr (Y.-J.R.); 2 Department of Orthopedic Surgery, Eulji University Hospital, Daejeon 35233, Korea; gywnsos89@naver.com; 3 Department of Orthopedic Surgery, Asan Medical Center, University of Ulsan College of Medicine, Seoul 05505, Korea; osdoc.koh@gmail.com (K.H.K.); jeonchoi@gmail.com (I.-H.J.)

**Keywords:** total shoulder arthroplasty, brachial plexus block, analgesia, neurologic exam

## Abstract

A continuous interscalene brachial plexus block (CIBPB) is usually administered before surgery in awake patients. However, the use of CIBPB before surgery could hinder the identification of nerve injuries after total shoulder arthroplasty (TSA). This study aimed to compare the analgesic effects of preoperatively and postoperatively initiated CIBPBs in patients undergoing TSA. The medical records of patients who underwent TSA between January 2016 and August 2020 were retrospectively reviewed. The following analgesic phases were used: intravenous (IV) patient-controlled analgesia (PCA) phase (IV PCA group, *n* = 40), preoperative block phase (PreBlock group, *n* = 44), and postoperative block phase (PostBlock group, *n* = 33). The postoperative initiation of CIBPB after a neurologic exam provided better analgesia than IV PCA and had no differences with the preoperative initiation of CIBPB, except for the worst pain at the postanesthetic care unit. Opioid consumption was significantly greater in the IV PCA group, but there were no differences between the PreBlock and PostBlock groups on operation day after the transfer to the general ward. The initiation of CIBPB after a patient’s emergence from general anesthesia had comparable analgesic efficacy with preoperative CIBPB but offered the chance of a postoperative neurologic exam.

## 1. Introduction

Multimodal analgesia has been strongly recommended in surgical patients to reduce opioid consumption [1]. In a multimodal analgesia strategy, regional anesthesia, including neuraxial anesthesia and peripheral nerve block, plays an important role. For shoulder arthroplasty, the interscalene brachial plexus block is a gold-standard regional anesthetic technique and has been performed by many anesthesiologists [2]. 

Peripheral nerve blocks, including the interscalene brachial plexus block, are usually administered before surgery in awake patients. It is widely assumed that preoperative peripheral nerve blocks cause preemptive analgesia [3], which is applied before noxious stimuli arise and could theoretically reduce persistent postoperative pain more effectively than conventional analgesic methods by inhibiting central sensitization [4]. However, some studies reported that peripheral nerve blocks did not cause preemptive analgesia in postoperative pain control [5,6]. Moreover, the application of a peripheral nerve block before surgery could hinder the identification of newly developed nerve injuries after shoulder arthroplasty. Although neurologic complications have been reported to be relatively uncommon, early diagnosis and management are considered important prognostic factors [7]. Therefore, some surgeons are reluctant to initiate analgesia by using interscalene brachial plexus blocks before the postoperative neurologic examination. 

In our center, the analgesic protocol in patients with total shoulder arthroplasty (TSA) was changed from intravenous (IV) patient-controlled analgesia (PCA) using fentanyl, to a continuous interscalene brachial plexus block (CIBPB) with preoperative initiation, and to CIBPB with postoperative initiation after the neurologic exam. Therefore, we aimed to compare the analgesic effects of each analgesic protocol by comparing the opioid consumption of patients undergoing TSA. In addition, we also analyzed the postoperative pain scores and the incidence of postoperative nausea and vomiting (PONV). 

## 2. Methods

This study was approved by the Institutional Review Board of our center (protocol no. 2020-1670; approval date: 11 November 2020), and written informed consent was waived owing to the retrospective nature of this study. 

### 2.1. Study Population

**Figure 1:**
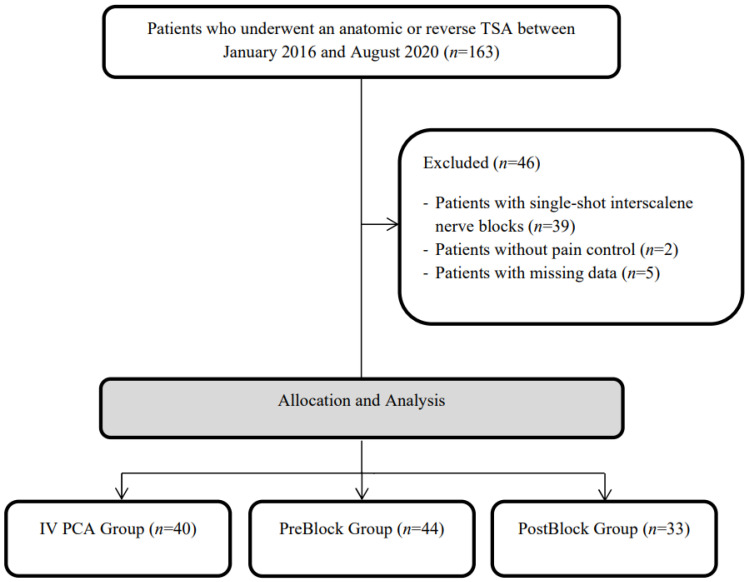
Flow diagram of this study.

The medical records of patients who underwent an anatomic or reverse TSA between January 2016 and August 2020 in our institution were retrospectively reviewed. A total of 163 consecutive TSA cases were identified in the electronic medical record system. All surgeries were performed at a tertiary center in Seoul, Korea. A total of 39 cases with single-shot interscalene nerve blocks were excluded. Two and five cases without pain control and with missing data about postoperative pain scores were also excluded, respectively (Figure 1). 

The analgesic method for patients undergoing TSA changed over time, and the study period could be divided into four phases: the IV PCA phase (IV PCA group, before February 2018), which involves only IV PCA; the single-shot interscalene block phase (between March 2018 and December 2019), which involves single-shot interscalene brachial plexus block without catheterization; the preoperative block phase (PreBlock group, between November 2018 and January 2020), which involves preoperative CIBPB with catheter insertion; and the postoperative block phase (PostBlock group, after January 2020), which involves postoperatively initiated interscalene brachial plexus block via a preoperatively inserted catheter. Among the four phases, the single-shot interscalene block phase was excluded to reduce the confounder in the analysis of the results. The numbers of patient in the IV PCA group, PreBlock group, and PostBlock group were 40, 44, and 33, respectively.

### 2.2. Anesthetic and Analgesic Methods

After the patients arrived at the operating room or the preanesthesia block room, standard monitoring including noninvasive blood pressure, heart rate, and oxygen saturation was performed. Thereafter, an ultrasound-guided interscalene brachial plexus block was administered to the PreBlock and PostBlock groups. For the CIBPB, the patients were placed in a lateral decubitus position, and the ipsilateral neck region was sterilized with betadine. A high-frequency linear probe (8–15 Hz) from an ultrasound machine (SONIMAGE HS1, Konica Minolta, Tokyo, Japan) was used and placed on the interscalene groove to find the brachial plexus between the anterior scalene muscle and middle scalene muscle. The depth and gain were finely adjusted to optimize the ultrasound view. The skin was infiltrated with 1 mL of 2% lidocaine, and an 18-gauge introducer needle connected to a 0.2%–0.3% ropivacaine infusion was inserted toward the brachial plexus (Sonolong sono or e-cath, Pajunk, Geisingen, Germany). When the needle tip reached between the C5 and C6 roots, 10–15 cc of 0.2%–0.3% ropivacaine was injected to the PreBlock group, whereas 2–3 cc normal saline was injected to the PostBlock group to confirm the location of the needle and secure the space for catheter insertion. After the injection of ropivacaine or normal saline, the prepared catheter was inserted under ultrasound guidance and was fixed firmly on the skin with skin adhesives. Thereafter, the patient was turned to a supine position, and general anesthesia was induced. In the IV PCA group, the patient received general anesthesia without any peripheral nerve block. In all groups, general anesthesia was induced using 1.5–2.5 mg/kg propofol. When the patients lost consciousness, desflurane or sevoflurane and 0.6–0.8 mg/kg rocuronium were administered to the patients, and the lungs were bag-mask ventilated. After reaching the adequate anesthetic depth, orotracheal intubation was performed. In all patients, an arterial cannula was inserted, and arterial blood pressure was monitored during the surgery. When the surgery was finished, the administration of anesthetic agents was halted, and the patients were transferred to the postanesthesia care unit (PACU).

**Figure 2:**
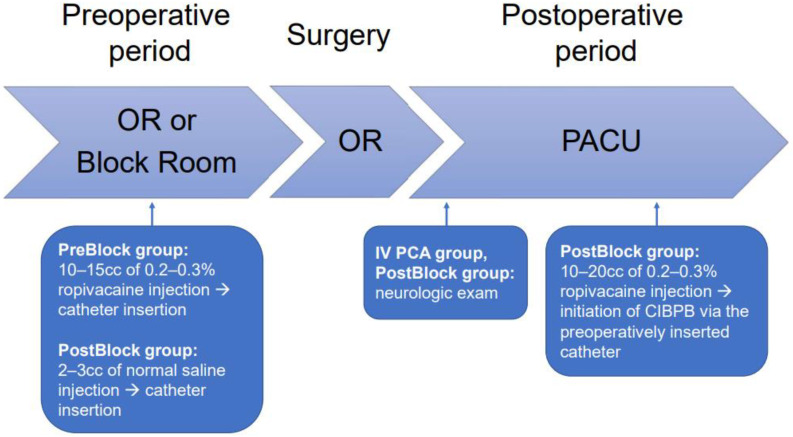
The strategy of postoperative analgesia. OR, Operating room; PACU, Postanesthetic care unit.

IV PCA was prepared with fentanyl citrate in normal saline and was started at the PACU. After the patients in the IV PCA group were transferred to the general ward, they were encouraged to use IV PCA for analgesia, and intravenous tridol or opioid such as hydromorphone was usually administered for the breakthrough pain. The nerve block PCA in the PreBlock and PostBlock groups was prepared using a mixture of ropivacaine and normal saline. In the PreBlock group, nerve block PCA was started as soon as the patient arrived at the PACU. In the PostBlock group, a bolus dose of 10–20 cc of 0.2–0.3% ropivacaine was injected via the preoperatively inserted interscalene catheter after the neurologic exam at the PACU, followed by the initiation of the nerve block PCA (Figure 2). Patients with nerve block PCA were recommended to use nerve block PCA for analgesia, and celecoxib and tapentadol were prescribed regularly. For the uncontrolled breakthrough pain, intravenous hydromorphone was administered. 

### 2.3. Surgical Procedures

A standardized deltopectoral approach was used in a beach chair position. The subscapularis was divided medially to the bicipital groove and was tagged with no. 5 Ethibond for subsequent repairs. After the humeral head was resected and capped with a protector, the glenoid was prepared. The glenoid surface was exposed after the posterior dislocation of the proximal humerus to allow anteroinferior capsule release. After dislocation, the arm position was maintained as neutral as possible to reduce tension to the brachial plexus. 

The labrocapsular complex was removed to achieve sufficient exposure and to position the glenoid baseplate. In this procedure, the inferior capsule was released along the glenoid neck to minimize injury to the axillary nerve. Glenoid reaming following the provisional central pin was applied to achieve the planned baseplate position. The base plate was placed on the prepared glenoid surface. 

On the basis of the arthroplasty system (reverse total shoulder or anatomical), the base plate position was changed. Furthermore, in anatomical arthroplasty, a metal-back or conventional system was used depending on the surgeons’ decision. If anatomical arthroplasty was performed using a reverse or metal-back system, a peripheral screw was inserted in the direction of the coracoid, inferior, and scapular spine. In conventional anatomical arthroplasty, a cemented polyethylene glenoid baseplate was applied. 

After the glenoid was prepared and the implant inserted, the humeral medullary canal was reamed until it was at the measured size of the stem. The stem trial was inserted and reduced temporally for testing stability and mobility. Before the real implant was inserted, a transosseous hole was made for the repair of the subscapularis tendon. If possible, transosseous subscapularis refixation was performed using no. 5 Ethibond.

### 2.4. Neurologic Exams

When the patient arrived at the PACU, a neurologic exam was performed to evaluate the function of the brachial plexus. Each nerve was evaluated as follows: C5, the anterolateral shoulder and proximal lateral upper arm; C6, the lateral upper arm, lateral forearm, first digit, radial aspect of the second digit; C7, the posterior upper arm, forearm to third digit, ulnar aspect of the second digit, and radial aspect of the fourth digit; C8, the dorsomedial upper arm, forearm to the fifth and ulnar aspect of the fourth digit, and ulnar border of the palm; and T1, the medial aspect of the elbow. Thereafter, the patient was asked to take the following actions to assess the motor function of each nerve: arm elevation for C5, elbow flexion for C6, elbow extension for C7, and finger spreading for C8. 

## 3. Outcome Measurements

The primary outcome of this study was total opioid consumption after TSA on operation day, postoperative day 1, and postoperative day 2. The opioids used on each day were summed and converted into a morphine equivalent dose. The worst and resting pain scores were investigated using a numerical rating scale (NRS) in the PACU and during postoperative day 2 in the general ward. Moreover, the incidence of newly developed neurologic deficits, PONV, use of rescue antiemetics, and desaturation events at the PACU were also obtained. 

## 4. Statistics

Baseline characteristics and preoperative data were compared among the three groups. All data were expressed as mean ± standard deviation or median (interquartile range (IQR)). Continuous variables were analyzed using the ANOVA F-test or the Kruskal–Wallis test. Post hoc analysis by Dunn’s test was performed to compare the groups. Categorical variables were analyzed using Fisher’s exact test or the chi-squared test, and Bonferroni correction was used for post hoc comparison. A *p*-value < 0.05 was considered statistically significant. All statistical analyses were performed using SAS version 9.4 (SAS Institute Inc., Cary, NC, USA).

## 5. Results

A total of 117 patients who underwent anatomical TSA or reverse TSA were included in the final analysis. Table 1 showed the baseline characteristics of the patients in each group. There were differences in age and surgical strategy. The other characteristics were not different.

Table 2 presents the opioid consumption, which is the primary outcome of this study. Opioid consumption at each time point showed a significant difference in the Kruskal–Wallis test. Post hoc comparison demonstrated that opioid consumption at the PACU was higher in the IV PCA and PostBlock groups than in the PreBlock group (all *p* < 0.001). Opioid consumption on operation day after transferring to the general ward was lower in the PreBlock and PostBlock groups than in the IV PCA group, although the PreBlock and PostBlock groups showed no differences. There were significant differences in opioid consumption on postoperative day 1 (POD1) and postoperative day 2 (POD2). The PreBlock and PostBlock groups showed lower opioid use than the IV PCA group, and the opioid consumption in the PostBlock group was lower than that in the PreBlock group.

Table 3 shows the worst and resting pain scores of each group. The worst NRS at the PACU demonstrated significant differences in the three groups, and the PreBlock group had the lowest NRS score, followed by the PostBlock group and IV PCA group. Although the resting NRS of the PreBlock group and PostBlock group at the PACU showed no differences, the resting NRS at the PACU was lower in these two nerve block groups than in the IV PCA group. The worst pain on the day of surgery at the general ward was lower in the PreBlock group and PostBlock group than in the IV PCA group, and the two nerve block groups showed no differences. The worst pain on POD1 demonstrated the same results. However, there were no difference in the worst pain on POD2 among all groups.

Neurologic deficits after TSA developed in four patients (3.4%): two patients in the IV PCA group and two patients in the PreBlock group (Table 3 and Table 4). The PONV in each group showed significant differences. The PreBlock and PostBlock groups did not have a different incidence of PONV, but the occurrences of PONV in these two groups were significantly less than those in the IV PCA group. Desaturation at the PACU was not different among the three groups.

## 6. Discussion

In this study, we found that the postoperative initiation of CIBPB via a preoperatively inserted catheter after a neurologic exam provided better analgesia than IV PCA. Furthermore, postoperatively initiated CIBPB was no different to preoperatively initiated CIBPB in pain score, except for reports of the worst pain at the PACU in patients undergoing TSA. Postoperatively initiated CIBPB showed better outcomes on POD 1 and POD2 in terms of opioid consumption. In addition, PONV was reduced in the nerve block groups compared with the IV PCA group.

An important finding of this study was that the two continuous nerve block groups regardless of CIBPB initiation timing had less opioid consumption and a lower NRS than the IV PCA group. This result was consistent with those of studies that identified the efficacy of CIBPB in shoulder surgery [8,9]. Furthermore, our study was the first trial that compared the efficacy of CIBPB according to initiation timing and proved that the postoperative initiation of CIBPB provided sufficient analgesia, similar to the preoperative initiation of CIBPB. The PostBlock group showed significant differences with the PreBlock group in opioid consumption and NRS score at the PACU, and this finding was an inevitable and expected consequence of the timing of the neurologic exam and the onset time of ropivacaine. The PostBlock group showed less postoperative opioid consumption than the PreBlock group in POD1, thus indicating that the PostBlock group provided better postoperative analgesia than the PreBlock group. However, this result could be explained by the change in the routine postoperative analgesic regimen at our institution. The amount of routinely prescribed opioid in the PreBlock phase was greater than that in the PostBlock phase. During the PreBlock phase, tapentadol 50 mg was prescribed to be administered orally twice a day, but after 1/3 of the PostBlock phase, tapentadol 50 mg was prescribed once a day. In addition, the lower opioid consumption in the PostBlock group might result from the longer-lasting effect of the ropivacaine bolus. Therefore, we suggest that CIBPB should be considered regardless of the initiation timing of the block to provide effective analgesia in patients undergoing shoulder surgery. Moreover, our study results demonstrated that the postoperative initiation of the CIBPB can further provide an additional benefit of immediate postoperative neurologic examination with equivalent postoperative analgesia compared with a conventional preoperatively initiated CIBPB.

The prevalence of nerve injury after shoulder arthroplasty was reported to be 1.2% to 19%, based on the diagnostic method [10,11]. The disparity of the incidence was derived from the definition of the nerve injury. However, many articles commonly showed that the neurologic deficits were usually transient and spontaneously resolved with conservative treatment alone [12,13]. However, it usually takes 3–6 months for neurologic symptoms to recover, and some patients with nerve injury may require diagnostic tests and/or additional surgical interventions [11,13]. In the current study, four patients developed neurologic deficits: two in the IV PCA group and two in the PreBlock group. Fortunately, two patients recovered from the neurologic deficits in a few weeks, while it took 6–7 months to recover from the symptoms in the other two patients. Furthermore, there were reports of permanent neurologic deficits due to the delays in diagnosis and loss of opportunity in treating the nerve injury [7]. To reduce nerve injury, intraoperative nerve monitoring during arthroplasty is performed in some centers. However, intraoperative monitoring is not always available, and the effect of real-time intraoperative nerve monitoring on the prevention of nerve injury is still questionable [14]. Therefore, the early detection of neurologic injury is crucial in the immediate postoperative period following shoulder arthroplasty. 

Most iatrogenic nerve injuries after TSA result from improper patient positioning or the traction of a nerve for surgical exposure and humeral distalization [10,12,15]. In case of nerve irritation due to the insertion of an instrument during surgery, the adjustment of the instrument position or the removal of the instrument could resolve neurologic symptoms and improve outcomes [16]. Therefore, a detailed neurologic examination that was performed after emergence from general anesthesia in the PACU immediately after surgery could be helpful. If the patients show abnormality in the postoperative neurologic exam, the position of the ipsilateral arm should be fixed with splinting, and close observation of the neurologic deficit should be performed [17]. A study reported that the most commonly injured nerve was the axillary nerve, followed by the radial nerve, median nerve, musculocutaneous nerve, and suprascapular nerve [12]. The sensory and motor functions of the brachial plexus could be altered by the administered local anesthetics after the interscalene brachial plexus block. A preoperatively performed interscalene block can mask the symptom of newly developed nerve injuries by blocking the nerves. Moreover, given that there is no definite method to reverse the effect of local anesthetics, immediate neurologic examination is unavailable in patients who received a preoperatively performed interscalene block regardless of whether it was a single-shot interscalene block or a CIBPB. On the contrary, the postoperative initiation of CIBPB not only makes immediate postoperative neurologic examination possible in the PACU but also showed a considerable analgesic effect that is comparable to that of the preoperative interscalene block. 

Peripheral nerve injury can possibly occur because of the interscalene brachial plexus block [18,19]. Even though an article showed that the placement of an interscalene block did not increase the risk of peripheral nerve injury after TSA, the possibility of neurologic injury after block placement still exists [20]. When a preoperative interscalene brachial plexus block is used, it may make it difficult to determine the cause of neurologic deficits even after the preoperative bolus effect wears off. Therefore, the postoperative initiation of CIBPB can be considered for postoperative pain control in TSA patients that have a high risk of nerve injury.

This study also had some limitations. First, there were inevitable flaws owing to its retrospective before-and-after study design with a relatively small sample size. Patients in the two nerve block groups underwent surgeries in a relatively recent period compared with the patients in IV PCA group. There may have been improvements in medical and surgical services, and these improvements possibly affected the results of our study. In addition, the routine postoperative analgesic regimen also changed over time. However, the overall postoperative managements were not meaningfully different between the two nerve block groups, and the difference in opioid consumption seemed not to be clinically important. Thus, we suggested that the initiation of CIBPB after neurologic evaluation is preferable as an effective analgesic method with minimal concerns on neurologic deficits. A further well-designed large-scale prospective study is necessary to confirm the analgesic effect of the initiation timing of CIBPB on postoperative pain after TSA. Second, there was a significant difference in the type of surgery among the three groups. The proportion of reverse TSA has been increasing. However, studies have demonstrated that patients undergoing reverse TSA or anatomical TSA have similar postoperative pain scores [21]. Thus, we assumed that the difference in surgery type did not influence our study results. 

## 7. Conclusions

The postoperative initiation of CIBPB after emergence from general anesthesia has comparable analgesic efficacy with the preoperative initiation of CIBPB in patients undergoing TSA. In addition, the postoperative initiation of CIBPB can lead to the early recognition of newly developed neurologic deficits.

## Figures and Tables

**Table 1:** Baseline characteristics.

	IV PCA Group (*n* = 40)	PreBlock Group (*n* = 44)	PostBlock Group (*n* = 33)	*p*-Value
Demographic Data				
Sex, female/male	34/6 (85.0/15.0)	36/8 (81.8/18.2)	24/9 (72.7/27.3)	0.432
Age (years)	73.33 ± 8.98	71.18 ± 9.03	76.21 ± 5.30	0.030
Height (cm)	151.17 ± 8.29	154.02 ± 7.28	153.48 ± 6.05	0.183
Weight (kg)	56.63 ± 9.81	58.48 ± 9.67	60.24 ± 9.52	0.286
Body mass index (kg/m^2^)	24.75 ± 3.68	24.64 ± 3.69	25.52 ± 3.49	0.533
Preoperative Medical History				
ASA PS *, 2/3/4	36/4/0 (90.0/10.0/0.0)	35/8/1 (79.5/18.2/2.3)	25/8/0 (75.8/24.2/0.0)	0.288
Hypertension	27 (67.5)	29 (65.9)	25 (75.8)	0.624
Diabetes mellitus	11 (27.5)	11 (25.0)	4 (12.1)	0.248
Coronary artery disease	4 (10.0)	5 (11.4)	4 (12.1)	>0.999
Cerebrovascular accident	3 (7.5)	3 (6.8)	6 (18.2)	0.240
Diagnosis				0.300
Cuff tear arthropathy	20 (50.0)	28 (63.6)	16 (48.5)	
Osteoarthritis	16 (40.0)	9 (20.5)	13 (39.4)	
Etc.	4 (10.0)	7 (15.9)	4 (12.1)	
Preoperative numerical rating score in the shoulder	5.50 (4.00, 7.00)	6.00 (5.00, 8.00)	6.00 (5.00, 8.00)	0.618
Surgical Data				
Surgical strategy				0.003
Anatomical total shoulder arthroplasty	18 (45.0)	7 (15.9)	5 (15.2)	
Reverse total shoulder arthroplasty	22 (55.0)	37 (84.1)	28 (84.8)	
Operation time (min)	163.43 ± 49.98	156.41 ± 35.54	158.94 ± 31.95	0.724

* ASA PS, American Society of Anesthesiologist Physical Status. Data are presented as mean ± standard deviation, median (interquartile range), or number (%).

**Table 2:** Postoperative opioid consumption.

	IV PCA Group	PreBlock Group	PostBlock Group	*p*-Value	Post Hoc Comparison		
Morphine Equivalent Dose	(*n* = 40)	(*n* = 44)	(*n* = 33)		IV PCA Group vs. PreBlock Group	IV PCA Group vs. PostBlock Group	PreBlock Group vs. PostBlock Group
Operation day (at the PACU *)	5.00 (3.00, 10.00)	0.00 (0.00, 5.00)	5.00 (4.00, 10.00)	<0.001	<0.001	0.866	<0.001
Operation day (at the GW ^†^)	17.00 (10.52, 25.30)	8.00 (8.00, 8.00)	8.00 (8.00, 8.00)	<0.001	<0.001	<0.001	0.837
Postoperative day 1	26.40 (20.32, 35.78)	16.00 (10.00, 16.00)	8.00 (8.00, 16.00)	<0.001	<0.001	<0.001	0.047
Postoperative day 2	21.70 (4.00, 30.34)	16.00 (10.00, 16.00)	8.00 (8.00, 8.00)	<0.001	0.081	<0.001	0.006

* PACU, Postanesthetic care unit; ^†^ GW, General ward. Data are presented as median (interquartile range).

**Table 3:** Postoperative pain and other clinical outcomes.

	IV PCA Group	PreBlock Group	PostBlock Group	*p*-Value	Post Hoc Comparison		
Numerical Rating Scale	(*n* = 40)	(*n* = 44)	(*n* = 33)		IV PCA Group vs. PreBlock Group	IV PCA Group vs. PostBlock Group	PreBlock Group vs. PostBlock Group
Worst pain at PACU *	6.00 (5.00, 7.00)	2.00 (0.00, 4.25)	5.00 (4.00, 6.00)	<0.001	<0.001	0.007	0.002
Resting pain at PACU *	3.00 (2.75, 4.00)	1.50 (0.75, 2.00)	2.00 (1.00, 3.00)	<0.001	<0.001	<0.001	0.078
Worst pain at GW ^†^ on operation day	5.00 (4.00, 6.00)	4.00 (3.00, 5.00)	4.00 (3.00, 5.00)	<0.001	0.001	0.001	0.906
Resting pain at GW ^†^ on operation day	3.00 (2.00, 3.00)	3.00 (1.00, 3.00)	2.00 (1.00, 3.00)	0.073			
Worst pain on postoperative day 1	5.00 (3.00, 5.00)	3.00 (3.00, 5.00)	3.00 (2.00, 4.00)	0.003	0.014	0.005	0.502
Resting pain on postoperative day 1	3.00 (3.00, 3.00)	3.00 (2.75, 3.00)	2.00 (1.00, 3.00)	0.001	0.510	0.001	0.004
Worst pain on postoperative day 2	3.00 (3.00, 5.00)	3.00 (3.00, 3.25)	3.00 (3.00, 5.00)	0.170			
Resting pain on postoperative day 2	3.00 (3.00, 3.00)	3.00 (1.75, 3.00)	2.00 (1.00, 3.00)	0.008	0.107	0.006	0.206
Other Clinical Outcomes							
Postoperative nausea and vomiting	16 (40.0)	3 (6.8)	3 (9.1)	<0.001	0.001	0.006	>0.999
Rescue antiemetics	0.00 (0.00, 1.00)	0.00 (0.00, 0.00)	0.00 (0.00, 0.00)	0.861			
Desaturation at the PACU *	7 (17.5)	4 (9.1)	3 (9.1)	0.488			
Neurologic deficits	2 (5.0)	2 (4.5)	0 (0.0)	0.553			

* PACU, Postanesthesia care unit; ^†^ GW, General ward. Data are presented as median (interquartile range) or number (%).

**Table 4:** Cases of newly developed neurologic deficits after total shoulder arthroplasty.

Case	Age (Year)	Sex	Surgical Strategy	Analgesic Strategy	Injured Nerve	Recovery
1	71	Female	Reverse TSA *	Intravenous patient-controlled analgesia	Musculocutaneous nerve, radial nerve	Improved in six months
2	75	Female	Anatomical TSA *	Intravenous patient-controlled analgesia	Axillary nerve, musculocutaneous nerve	Improved in three weeks
3	75	Female	Reverse TSA *	Preoperative continuous interscalene block	Musculocutaneous nerve, radial nerve	Improved in one week
4	65	Female	Reverse TSA *	Preoperative continuous interscalene block	Radial nerve	Improved in seven months

* TSA, Total shoulder arthroplasty.

## Data Availability

The data presented in this study are available on request from the corresponding author. The data are not publicly available due to conditions of the ethics committee of our university.
